# Assessing the overuse of antibiotics in children in Saudi Arabia: validation of the parental perception on antibiotics scale (PAPA scale)

**DOI:** 10.1186/1477-7525-11-39

**Published:** 2013-03-11

**Authors:** Arwa Alumran, Xiang-Yu Hou, Cameron Hurst

**Affiliations:** 1 School of Public Health & Social Work, Queensland University of Technology, Victoria Park Rd, Kelvin Grove, Brisbane, QLD 4059, Australia; 2 Institute of Health and Biomedical Innovation, Queensland University of Technology, Brisbane, Australia; 3 Health Information Management and Technology Department, College of Applied Medical Sciences, University of Dammam, Dammam, Saudi Arabia

**Keywords:** Antibiotics overuse, Psychosocial, Measurement instrument, Reliability, Validity, Exploratory factor analysis, Saudi Arabia

## Abstract

**Background:**

Antibiotics overuse is a global public health issue influenced by several factors, of which some are parent-related psychosocial factors that can only be measured using valid and reliable psychosocial measurement instruments. The PAPA scale was developed to measure these factors and the content validity of this instrument was assessed.

**Aim:**

This study further validated the recently developed instrument in terms of (1) face validity and (2) construct validity including: deciding the number and nature of factors, and item selection.

**Methods:**

Questionnaires were self-administered to parents of children between the ages of 0 and 12 years old. Parents were conveniently recruited from schools’ parental meetings in the Eastern Province, Saudi Arabia. Face validity was assessed with regards to questionnaire clarity and unambiguity. Construct validity and item selection processes were conducted using Exploratory factor analysis.

**Results:**

Parallel analysis and Exploratory factor analysis using principal axis factoring produced six factors in the developed instrument: knowledge and beliefs, behaviours, sources of information, adherence, awareness about antibiotics resistance, and parents’ perception regarding doctors’ prescribing behaviours. Reliability was assessed (Cronbach’s alpha = 0.78) which demonstrates the instrument as being reliable.

**Conclusion:**

The ‘factors’ produced in this study coincide with the constructs contextually identified in the development phase of other instruments used to study antibiotic use. However, no other study considering perceptions of antibiotic use had gone beyond content validation of such instruments. This study is the first to constructively validate the factors underlying perceptions regarding antibiotic use in any population and in parents in particular.

## Introduction

Although antibiotics are targeted to kill or inhibit the growth of bacteria and have no effect on viral agents [1], they are often inappropriately used to treat viral infections such as upper respiratory tract infections (URTIs). URTIs are usually self-limiting and resolve in the same amount of time regardless of antibiotic consumption [2]. Thus, using antibiotics to treat these viral infections is considered misuse or overuse of antibiotics. This misuse/overuse is common in children [3,4], and is currently considered to be one of the major worldwide public health issues [5-7].

Antibiotics misuse/overuse may cause several problems, for instance: development of antibacterial resistance [8,9], increasing the burden of chronic diseases and rising costs of health services [10], and the development of side effects (e.g. adverse gastrointestinal effects) [11].These adverse effects are more significant in children according to Simasek [12].

This misuse may be due to reasons related to: patients, parents or guardians, or the medical practitioner. Several studies have discussed the reasons associated with antibiotics overuse. These include: attitudes, beliefs, knowledge of antibiotic use [13-15], behaviours (e.g. over-the-counter medication and self-medication) [5,16,17], patients’ perceptions regarding patient-doctor interaction, patient satisfaction, and patients’ experience with antibiotics [15,18]. These studies have provided a framework for the development of the Parental Perception on Antibiotics Scale –‘The PAPA Scale’ [19].

The behaviours associated with the overuse of antibiotics may include antibiotics self-medication and over-the-counter medication. These behaviours specifically are considered a public health issue in many middle-east countries that are similar to Saudi Arabia, geographically and culturally [13,16,17,20]. However, information regarding trends in antibiotic consumption in Saudi Arabia is very limited. Moreover, there are no reports on parents’ behaviours regarding antibiotics use among Saudi children, especially those with upper respiratory tract infections (URTIs) [21]. As a result, it is important to measure this psychosocial phenomenon in Saudi Arabia.

In order to measure such psychosocial phenomena, a valid and reliable measurement instrument needs to be available [22]. Assessing the validity of an instrument involves confirming the instrument’s capability of measuring what it is intended to measure [23]. However, an extensive literature review has not revealed any validated instrument worldwide that measures the factors influencing antibiotics overuse in children with URTIs [24]. This study aims to validate a developed and content-validated instrument [19]; further validation includes construct validity of the instrument using factor analysis, which will determine the number and nature of the underlying construct in the developed instrument.

## Methods

This was a cross-sectional study design using a preliminary-validated questionnaire [19]. The required ethical approvals were obtained from Queensland University of Technology (ethical approval number: 1200000022) and the Ministry of Education in the Eastern Province in Saudi Arabia (ethical approval number: 33505889). The questionnaire was distributed to parents of children (younger than 12 years old) in primary schools in the Eastern Province of Saudi Arabia between March to April 2012. Participants’ consent was implied by the return of the completed questionnaire.

Only questionnaires completed by one of the parents or a legal guardian were included in the study. One questionnaire was excluded because it was completed by a sibling who was less than 18 years old.

### Instrument development

The PAPA scale was developed to assess parental perceptions regarding antibiotics. This scale aims to assess the factors influencing parents to use antibiotics for their children, especially in relation to upper respiratory tract infections. A content evaluation panel was developed to assess the content and face validity of the instrument by building a group brainstorming process [25]. The scale items were firstly derived from relevant literature in the field. This was followed by a three-round Delphi process conducted using a panel of experts knowledgeable in such areas as pediatrics, infectious diseases, epidemiology, family medicine, psychology and counseling, and social sciences. The report of this study has been published elsewhere [19].

Experts were provided with a pool of 80 questions retrieved from the relevant literature [18,26-29]. They were asked to choose the most relevant questions to measure the study objectives; i.e. factors influencing the overuse of antibiotics in children with URTIs in Saudi Arabia for the first round. Experts were also invited to generate ideas in this round [30]. The included questions from the first round were then sent again to the same panel members with the percentage of agreement for each item [31]. In this second round, experts were asked to agree, disagree, and/or comment on the items. The third and last round was sent to the experts to obtain their final confirmation on the instrument. The development process involved face validity as well as content validation. Face validity was conducted by asking the experts to comment on the clarity and flow of the questions in the proposed questionnaire.

A 58-question content-validated survey was developed to conduct this study [19]. The first part of the questionnaire, which is not the focus of this study, dealt with parental demographics and children’s health-related history. The second part consisted of questions about the factors associated with antibiotics use, e.g. parental knowledge, behaviours, attitudes and beliefs about antibiotics use for children younger than 12 years of age. The last part was to assess the face validity of the questionnaire. All questions relating to antibiotics use were measured on a five-point Likert scale. Questions to assess the face validity of the questionnaire were on Binary scale (yes/no).

### Statistical analysis

Personal characteristics were summarised using frequencies and percentages. The association of antibiotics use per year with the frequency of common cold episodes per year was assessed using cross tabulation.

Parallel analysis based on Principal Components Analysis was conducted; scree plots and Kaiser Criteria (Eigen value > 1) were used along a theoretical basis for choosing the number of factors. As this is a multivariate analysis, missing values were excluded listwise from the list.

Following the parallel analysis, Principal axis factoring was used to determine the nature of the underlying factors [25]. Both an orthogonal (Varimax) and an oblique (Promax) rotation were performed on the factor solution to determine which type of rotation was most suitable. The internal consistency of the instrument (i.e. reliability) was measured using Cronbach’s alpha.

All data analysis was conducted using the Statistics Package for Social Sciences (SPSS v19) with the exception of the parallel analysis, which used the *nFactor* library (v2.3.3) within the R statistics package (v2.14.2).

## Results

The questionnaires were completed by 238 parents (25% response rate). Mothers were more responsive than fathers; 70 percent of the parents in the study are mothers. Parents’ personal characteristics are summarised in Table 1. Some demographic differences were noticeable between mothers and fathers in the sample. With regard to age, the average age category of mothers in the study appears to be 31-40 years old (52%), while fathers tend to fall within the age category of 41-50 years old (44%). Most mothers and fathers are employed (56% and 79% consecutively), and third of mothers are housewives (34%). Moreover, illiteracy is more observable in mothers (2%), while all fathers in the study are literate. The majority of mothers and fathers have a diploma or a bachelor degree (76% and 55% consecutively). However, only 2 percent of mothers have higher degrees, compared to 26 percent of fathers.

**Table 1:** Demographic characteristics of study participants

**Variable**	**Mothers N = 167 (%)**	**Father N = 70 (%)**	**Total N = 237 (%)**
**Age**			
20 – 30	22 (13.2)	0 (0)	22 (9.3)
31 – 40	86 (51.5)	20 (28.6)	106 (44.7)
41 – 50	36 (21.6)	31 (44.3)	67 (28.3)
>50	0 (0)	15 (21.4)	15 (6.3)
**Missing**	23 (13.8)	4 (5.7)	27 (11.4)
No. of children*			
1 child	43 (26.1)	20 (8.6)	63 (27.0)
2 children	49 (29.7)	19 (8.2)	68 (29.2)
3 children	57 (34.5)	17 (7.3)	74 (31.8)
4 children	11 (6.7)	8 (3.4)	19 (8.2)
5 children	5 (3.0)	4 (1.7)	9 (3.9)
**Missing**	2 (1.2)	2 (2.9)	4 (1.7)
**Educational level**			
Illiterate	2 (1.2)	0 (0)	2 (0.9)
No formal education	2 (1.2)	0 (0)	2 (0.9)
Junior high school	7 (4.2)	7 (10.1)	14 (6.0)
High school	26 (15.8)	6 (8.7)	32 (13.7)
Diploma or bachelor	125 (75.8)	38 (55.1)	163 (13.7)
Higher degrees	3 (1.8)	18 (26.1)	21 (9.0)
**Missing**	2 (1.2)	1 (1.4)	3 (1.3)
**Employment**			
Unemployed	3 (1.8)	2 (2.9)	5 (2.1)
Employed	91 (55.5)	55 (78.6)	146 (62.4)
Student	6 (3.7)	0 (0)	6 (2.6)
Housewife	56 (34.1)	0 (0)	56 (23.9)
Self-employed	5 (3.0)	7 (10.0)	12 (5.1)
Retired	3 (1.8)	6 (8.6)	9 (3.8)
**Missing**	3 (1.8)	0 (0)	3 (1.3)
Monthly income**			
Low	12 (8.0)	9 (13.8)	21 (9.8)
Low middle	53 (35.3)	20 (30.8)	73 (34.0)
Middle	54 (36.0)	16 (24.6)	70 (32.6)
High middle	19 (12.7)	12 (18.5)	31 (14.4)
High	12 (8.0)	8 (12.3)	20 (9.3)
**Missing**	17 (10.2)	5 (7.1)	22 (9.3)
**Trained in health-related fields**			
Yes	27 (16.5)	10 (14.7)	37 (15.9)
No	137 (83.5)	58 (85.3)	195 (84.1)
**Missing**	3 (1.8)	2 (2.9)	5 (2.1)
**Geographical background**			
Eastern region	91 (59.5)	34 (55.7)	125 (58.4)
Western region	13 (8.5)	7 (11.5)	20 (9.3)
Middle region	23 (15.0)	10 (16.4)	33 (15.4)
Northern region	2 (1.3)	3 (4.9)	5 (2.3)
Southern region	24 (15.7)	7 (11.5)	31 (14.5)
**Missing**	14 (8.4)	9 (12.9)	23 (9.7)

*Children less than 12 years old.

** Income: low: <SR4000 (<$1066), Low middle: SR4000-11,999 ($1,066-3,199), Middle: SR12,000–21,999 ($3,199–5,866), High middle: SR22,000–34,999 ($5,866–9,332), High: > SR35,000 (> $9332).

Parents were asked to assess their child’s health-related history in relation to the number of common cold episodes per year and the number of antibiotic use per year (see Table 2). According to the parents, 13 (5.5%) children in the study had a serious infectious disease in the past including chicken pox and unidentified respiratory infections. Thirty-two (13.4%) children had chronic diseases such as heart disease, diabetes, asthma, and allergies.

**Table 2:** Children’s health-related history according to their parents

**Common cold episodes/year**	**Antibiotic usage/year**				
	**Never**	**Once**	**2 - 3 times**	**4–6 times**	**> 6 times**
Never	**7**	0	0	0	0
Once	7	**23**	12	2	0
2–3 times	13	24	**65**	9	1
4–6 times	2	6	12	**33**	4
> 6 times	0	0	3	2	**10**

Total = 235 (3 missing values).

**Figure 1:**
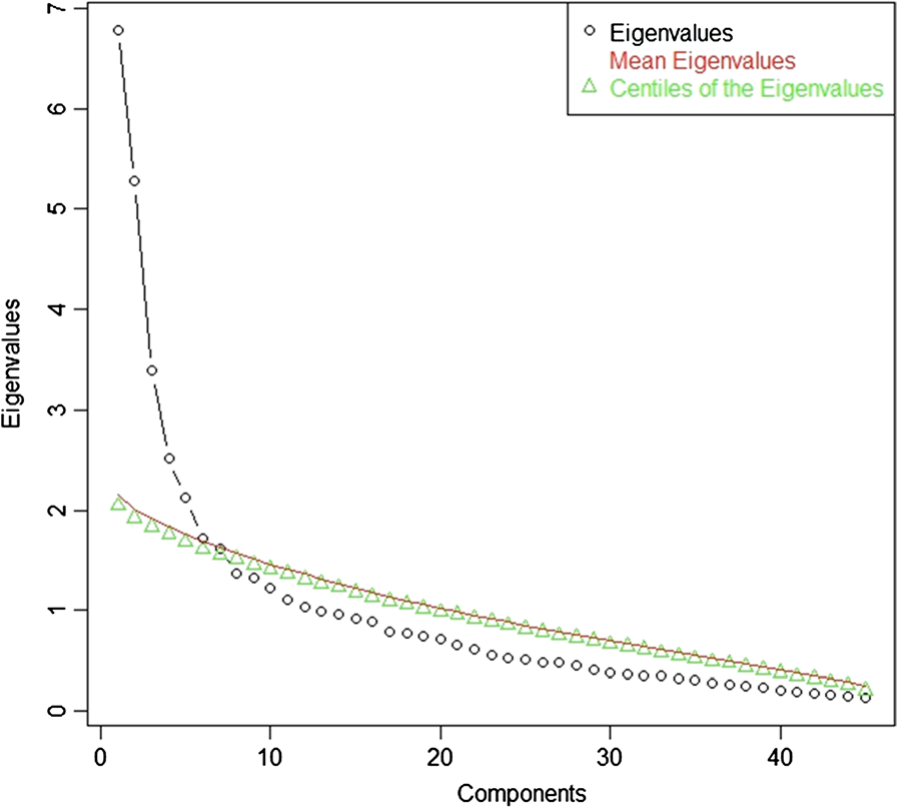
Parallel Analysis derived from a principal component analysis.

Parallel analysis was performed to decide on the number of factors to retain in the Parental Perception on Antibiotics Scale (PAPA Scale). Based on a 10,000-permutations parallel analysis, a six-factor solution was produced (Figure 1).

According to the parallel analysis, a six-factor solution was used. The coefficients in the pattern matrix in Table 3 show the number and nature of factors in the PAPA Scale using Exploratory factor analysis. The produced factors include: Factor 1: knowledge and beliefs (10 items), Factor 2: behaviours (6 items), Factor 3: sources of information (7 items), Factor 4: adherence (5 items), Factor 5: awareness about antibiotics resistance (5 items), and Factor 6: parents’ perception on doctors’ prescribing behaviours (3 items). In addition, the inter-factor correlation matrix shown in Table 4 suggests that at least some of the factors are moderately correlated indicating that an oblique measurement model is justified.

**Table 3:** The pattern coefficients from a principal axis factoring using promax rotation

**Factors and items**	**Items loadings**
**Factor 1: Knowledge and beliefs**	
**1-** Antibiotics are needed for: the common cold	.627
**2-** Antibiotics are needed for: sore throat	.631
**3-** Antibiotics treat viral infections	.461
**4-** Antibiotics can cure ALL types of infections (viral, bacterial, & fungal)	.574
**5-** When I visit the doctor for my child’s common cold I expect a prescription for medication including antibiotics	.594
**6-** Antibiotics are helpful in treating common colds among children	.687
**7-** Children with common colds get better faster when antibiotics are given	.749
**8-** In the past, antibiotics have cured my child’s cold symptoms	.628
**9-** My child will be sick for a longer time if he/she doesn’t receive an antibiotic for cough, cold, or flu symptoms.	.613
**10-** If my child has a cold or cough it is best to get an antibiotic to get rid of it	.672
**Factor 2: Behaviours:**	
**11-** Antibiotics should be sold without a prescription	443
**12-** In the past, I have stopped giving my child an antibiotic because my friends/family advised me to	.426
**13-** I get my child’s antibiotics from the pharmacy without a prescription	.809
**14-** I generally store antibiotics at home for when they are needed	.534
**15-** In the past, I have given my child an antibiotic without a prescription when he/she had a high temperature for a few days	.874
**16-** In the past, I have changed doctors when my doctor did not prescribe antibiotics for my child	.623
**Factor 3: Sources of information:**	
**17-** I get my health-related information from the pharmacist	.479
**18-** I get my health-related information from nurses and/or other allied health professionals	.472
**19-** I get my health-related information from books and/or scientific literature	.759
**20-** I get my health-related information from family and/or friends	.621
**21-** I get my health-related information from the internet	.773
**22-** I get my health-related information from the media: TV, radio, newspapers	.789
**23-** I get my health-related information from my previous experience	.556
**Factor 4: Adherence:**	
**24-** It is not important to follow antibiotics doses strictly	.465
**25-** Skipping one or two antibiotic doses doesn’t make much difference	.716
**26-** If my child gets better I can reduce the dose of antibiotics	.902
**27-** If my child’s condition is mild I would give the antibiotic according what I see is suitable for his/her condition	.517
**28-** In the past, I have stopped giving my child an antibiotic because he/she felt better	.697
**Factor 5: Awareness about antibiotics resistance**	
**29-** Antibiotics treat bacterial infections	.446
**30-** Antibiotics are generally safe	-.422
**31-** Antibiotics can be harmful to one’s health	.444
**32-** Some germs are becoming harder to treat with antibiotics	.446
**33-** Some germs can become resistant to antibiotics if they are taken in inadequate doses	.674
**Factor 6: Parent’s perception on doctors’ prescribing behaviors**	
**34-** I think doctors prescribe too many antibiotics	.441
**35-** Doctors don’t inform parents well about their child’s condition	.622
**36-** Doctors aren’t well informed about judicious antibiotics use	.674
**Items excluded due to only trivial loadings on all factors (coefficient < 0.4):**	
**37-** Antibiotics are needed for: ear infection	
**38-** If my child is asleep I will not wake him/her up for the dose of antibiotic	
**39-** In the past, I have stopped giving my child an antibiotic because he/she had side effects	
**40-** In the past, I have taken my child to a doctor when he/she had a high temperature for a few days	
**41-** I get my child’s antibiotics from the pharmacy with a prescription	
**42-** In the past, I have asked the doctor to prescribe medication for my child’s common cold	
**43-** I get my health-related information from my doctor	
**44-** When I visit the doctor for my child’s common cold and do not get antibiotics, I get dissatisfied	
**45-** Frequency of antibiotic use does not influence its effectiveness	

The likely construct associated with each factor is also included.

**Table 4:** Inter-factor correlation matrix

**Factor**	**1**	**2**	**3**	**4**	**5**	**6**
**1**	1.000	.038	-.058	.171	.010	-.078
**2**	.038	1.000	.113	.535	-.403	-.098
**3**	-.058	.113	1.000	.127	-.189	.192
**4**	.171	**.535**	.127	1.000	-.473	.104
**5**	.010	**-.403**	-.189	**-.473**	1.000	-.017
**6**	-.078	-.098	.192	.104	-.017	1.000

Extraction method: principal axis factoring.

Rotation method: promax rotation with Kaiser normalization.

By assessing the Instrument’s reliability it was found that the total and sub-scales were demonstrated as being reliable with the overall Cronbach’s alpha = 0.87, and the individual subscales Cronbach’s alphas ranging from 0.771 to 0.794.

## Discussion

After conducting parallel analysis and factor analysis to the newly developed and content validated measurement instrument, the following factors emerged from the analysis: knowledge and beliefs, behaviours, sources of information, adherence, awareness about antibiotics resistance, and parents’ perception on doctors’ prescribing behaviours.

The influencing factors on the overuse of antibiotics include psychosocial factors such as attitudes and beliefs; knowledge-related factors that may lead to unwanted behaviours such as parents’ pressure and inappropriate use of antibiotics; and demographic factors including education levels, socioeconomic status, and employment.

**Figure 2:**
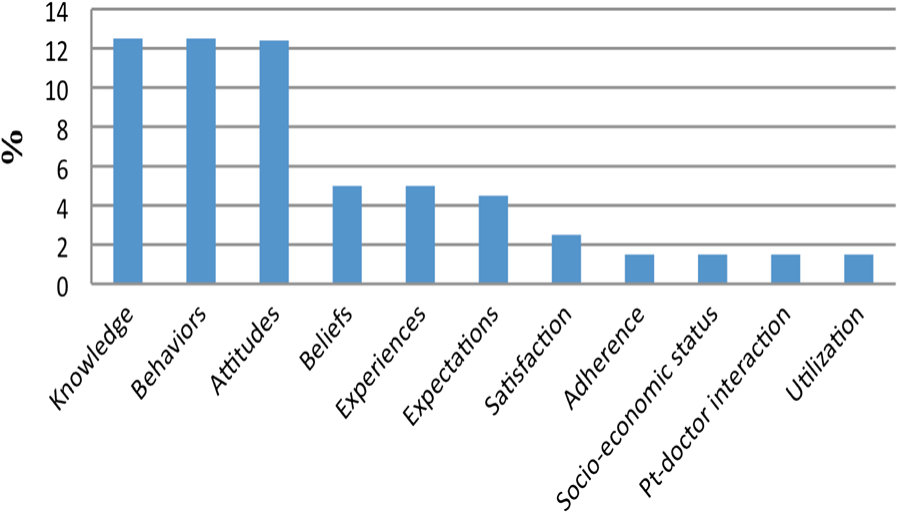
The frequency distribution of the dimensions mentioned in the literature.

**Figure 3:**
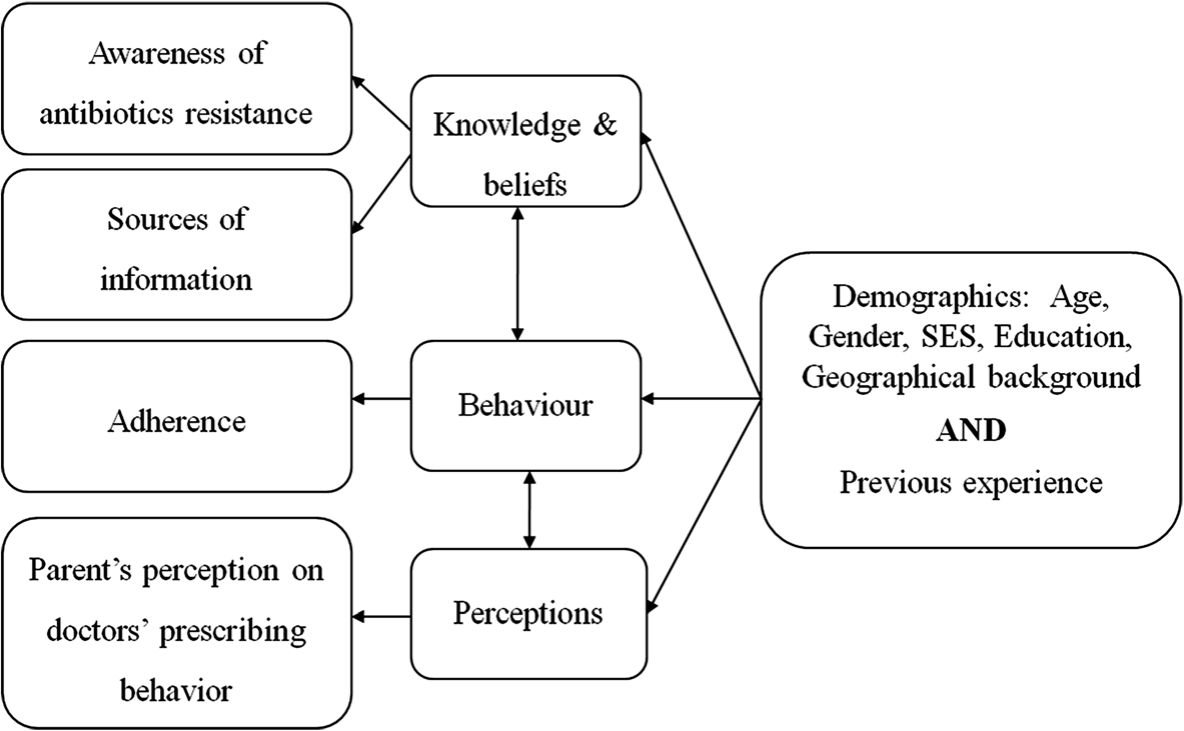
Conceptual framework.

The constructs measured in the literature in the field of antibiotics use include: attitudes, beliefs, knowledge (including experience with antibiotics), and behaviours (over-the-counter medication and self-medication) [5,13,16,17,20,32-35]. Other factors are measured within these major dimensions including: patient expectations and adherence to antibiotics, patients’ perceptions regarding patient-doctor interaction, and patient satisfaction [15,18,36,37]. Demographic characteristics were also measured in the reviewed literature relating to antibiotics use including: age, gender, level of education, and socio-economic status. Figure 2 shows the frequency of the dimensions reviewed in other studies that were conducted to measure the use of antibiotics. Thus, according to the dimensions available in previous studies and the dimensions present from this study, a conceptual model was created to show the relationship between the factors underlying antibiotics use/overuse (Figure 3).

Further psychometric testing is needed to determine the construct, concurrent, discriminate, and predictive validity of the PAPA scale. Moreover, the developed instrument can now be used in future research by translating it and culturally adapting it to different communities. In psychosocial research, instruments are frequently translated and cultural adapted to fit the population at hand [38]. Most instruments are developed in English. Therefore, for non-English speaking populations that are significantly different from the population used to develop the instrument, researchers usually translate and culturally adapt the English instrument to fit the local population [38]. Translation usually is a more efficient key for the scarcity of available instruments.

The PAPA scale produces a valid and reliable measurement instrument that can be used to assess parental perceptions regarding antibiotics. Since antibiotics overuse [3,4] and antibiotics resistance are global public health issues [8,9], many studies are targeted to minimise this problem. The PAPA scale could be effective in cross-sectional studies that aim to reduce the overuse of antibiotics in a community, starting by understanding the reasons behind this overuse. This, in turn, could inform the development of interventions directed to minimise the overuse of antibiotics.

### Limitations

The survey was distributed within primary schools in order to capture a more generalised cross-section of the community. However, since kindergartens are scarce in Saudi Arabia and not mandatory like primary, secondary, and high schools, parents of children under the age of six are not represented in such a sample unless there is more than one child in the household. Consequently, this could be considered a source of bias [39]. Also, another limitation is the low response rate which may raise concerns about selection bias.

## Conclusion

This is the first paper to validate an instrument that measures the overuse of antibiotics at the patients/parents level. The study shows promising results, producing evidence of strong collection of conceptually homogenous items and clear alignment of the ‘factors’ with constructs identified in early phase. This instrument now needs further validation such as: confirming the construct validity using confirmatory factor analysis, and criterion-related validity.

## Competing interest

There is no competing interest related to this study. Funding was received from University of Dammam, Saudi Arabia.

## Authors’ contributions

AA carried out the main intellectual contribution to the conception and design of the study, data collection, and made substantial contribution in the analysis and interpretation of the data. XH participated in the design of the study, and has been involved in revising the manuscript critically for important intellectual content. CH participated in the design of the study, made substantial contribution in the analysis and interpretation of the data, was involved in the revising the manuscript critically for important intellectual content, and has given the final approval of the version to be published. All authors read and approved the final manuscript.
